# Atypical pemphigus foliaceus with pustular lesions: a case series

**DOI:** 10.1186/s13256-025-05324-w

**Published:** 2026-06-24

**Authors:** Miranti Pangastuti, Annisa Sundani, Oki Suwarsa, Endang Sutedja, Chaerani Pratiwi Firdaus, Reti Hindritiani, Laila Tsaqilah, Hermin Aminah Usman, Maria Angela Putri Maharani, Fadhli Rajif Tangke, Nur Mala Il Ala

**Affiliations:** 1 Department of Dermatology and Venereology, Faculty of Medicine, Universitas Padjadjaran—Dr. Hasan Sadikin Hospital, Jl. Pasteur 38, Bandung, West Java 40161 Indonesia; 2 Department of Anatomical Pathology, Faculty of Medicine, Universitas Padjadjaran—Dr. Hasan Sadikin Hospital, Bandung, Indonesia; 3 Department of of Anatomical Pathology, Faculty of Medicine, Universitas Indonesia—Dr. Cipto Mangunkusumo Hospital, Jakarta, Indonesia

**Keywords:** Atypical pemphigus foliaceus, Case report, Pustular dermatosis, Vesiculobullous disease, Direct immunofluorescence

## Abstract

**Background:**

Pemphigus foliaceus usually presents as erosion, scales, and crusts on an erythematous base. Atypical presentations of pemphigus foliaceus, especially with pustular lesions, are exceedingly rare and pose significant diagnostic challenges. This case series highlights two cases of atypical pemphigus foliaceus with prominent pustular lesions that were encountered over 12 years.

**Case presentation:**

We report two cases of atypical pemphigus foliaceus in an 18-year-old Sundanese female patient and a 57-year-old Javanese male patient. Both patients had clinical manifestations of prominent pustular lesions, and histopathological examination revealed neutrophilic infiltration. However, direct immunofluorescence examination showed intercellular immunoglobulin G deposits on the surface of keratinocytes, which supported the diagnosis of pemphigus foliaceus. The lesions of the first patient showed improvement following the administration of cyclophosphamide 100 mg daily. The second patient, initially treated with sulfasalazine without success, showed improvement with the combination of corticosteroid equivalent to prednisone 1 mg/kg/day, azathioprine 50 mg twice daily, and psychotherapy.

**Conclusion:**

Pemphigus foliaceus can occur with atypical clinical manifestations, including prominent pustular lesions with neutrophilic infiltration, resembling pustular dermatosis’s clinical manifestations. However, direct immunofluorescence examination may help to differentiate atypical pemphigus foliaceus and other differential diagnoses. Given the challenges in diagnosing chronic vesiculobullous diseases with atypical presentations, clinicians should consider pemphigus foliaceus in the differential diagnosis of prominent pustular lesions to avoid misdiagnosis and ensure appropriate management.

## Background

Pemphigus foliaceus (PF) is a chronic autoimmune vesicobullous disease characterized by the presence of immunoglobulin (Ig) G antibodies against desmoglein 1 [1]. The incidence of PF is rare, with annual incidence in South Korea of 1.1 per 1 million population [2]. The typical clinical manifestations of PF include erosions, scales, and crusts on an erythematous base, along with histopathological features such as eosinophilic spongiosis and acantholysis [3, 4]. Atypical presentations with pustular lesions and neutrophilic infiltration are exceedingly rare and pose challenges in establishing the diagnosis. In 1996, Hoss *et al*.[3] reported PF with neutrophilic lesions for the first time. This case report aims to highlight two cases of atypical PF with pustular lesions and neutrophilic infiltrations encountered in our hospital in Indonesia over the past 12 years, enhancing the understanding of diagnosis in such rare presentations, which can be challenging for clinicians.

## Case presentation

### Case 1

**Fig. 1:**
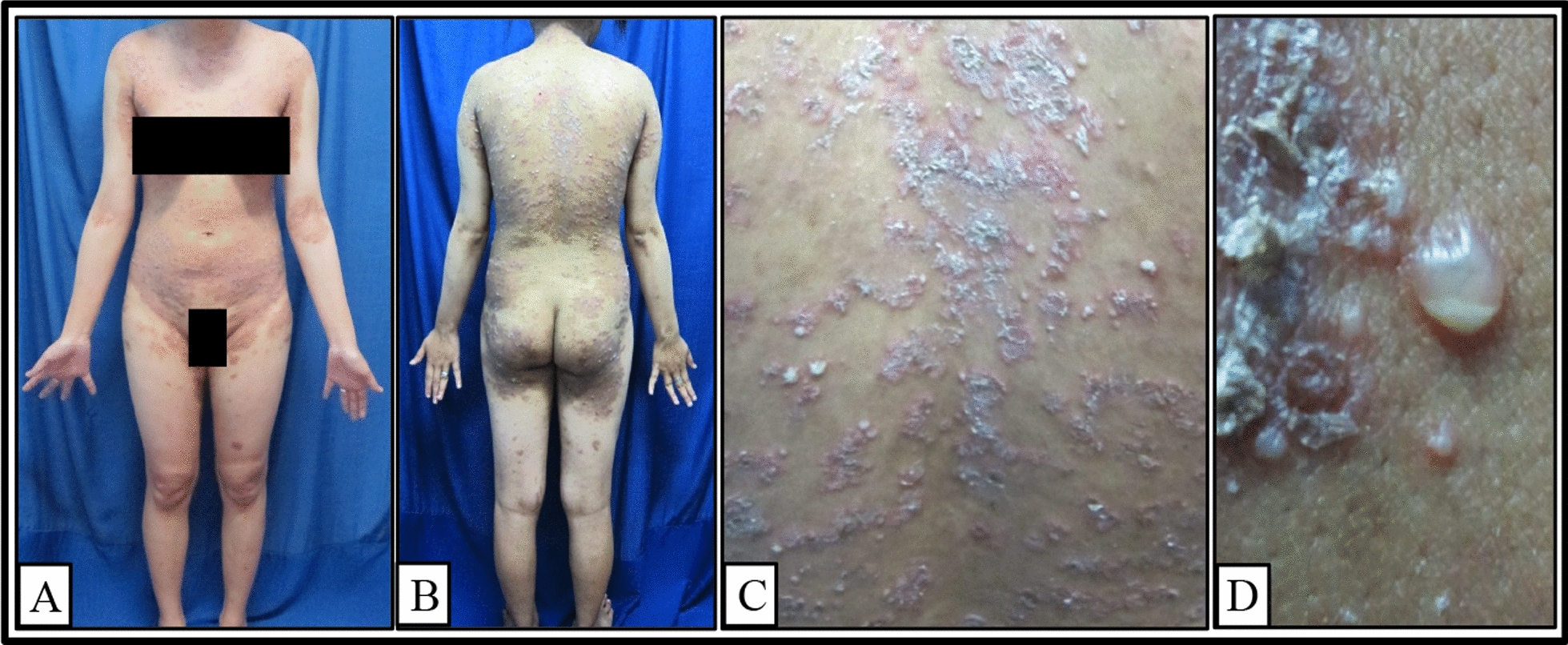
Patient 1: Skin lesions on the front view of the body (**A**), back view of the body (**B**), and back (**C**), and 'half–half' blister lesions on the back (**D**)

**Fig. 2:**
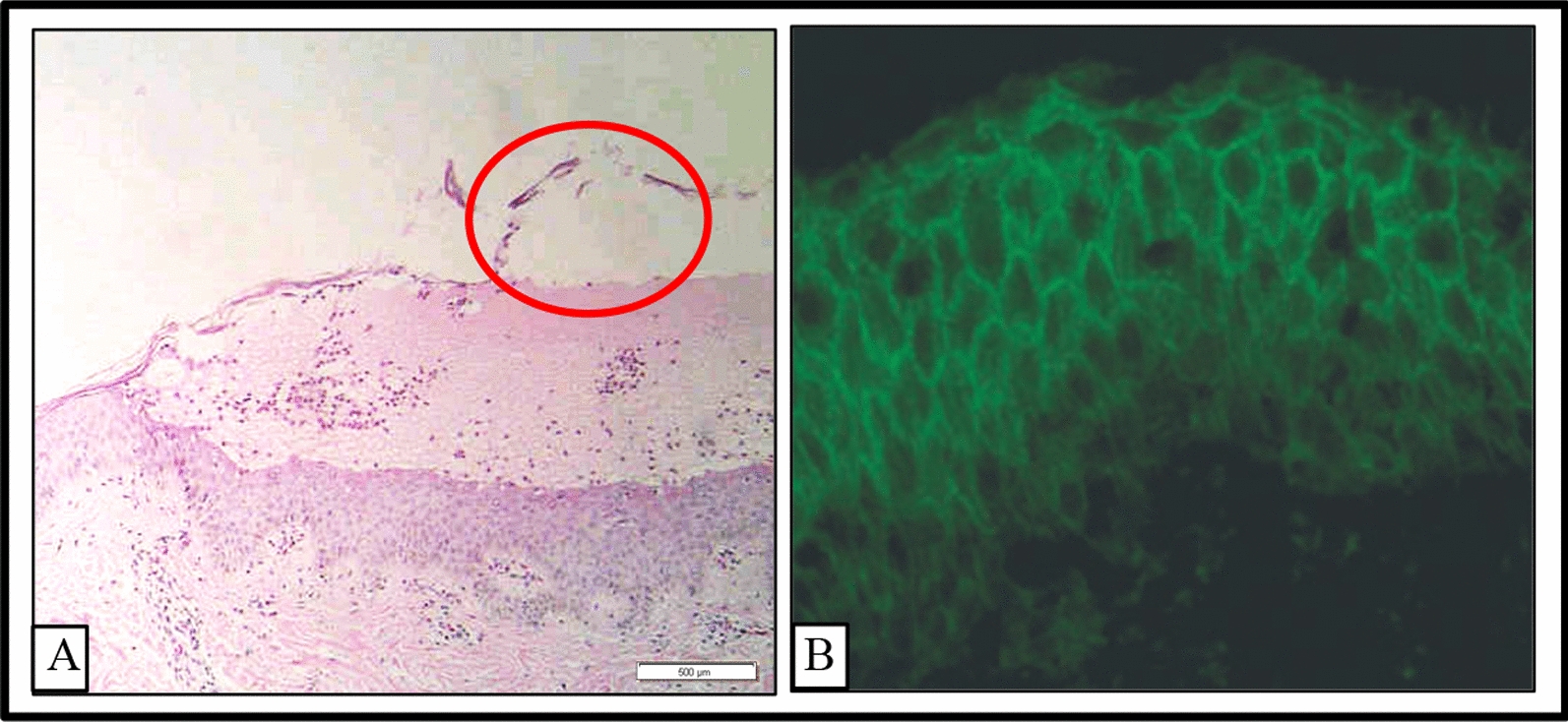
Patient 1: Histopathological examination results showing a subcorneal blister consisting of neutrophilic inflammatory cells (red circle) (**A**) and direct immunofluorescence examination results showing immunoglobulin G deposits with intercellular patterns in the epidermis (**B**)

An 18-year-old Sundanese female patient presented with itchy erythematous macules and pustules, which had been present for 6 years and progressively spread over the 3 months preceding the consult. The patient had been treated by several dermatologists and was given oral corticosteroid medication, but the skin disorder did not improve significantly. Physical examination revealed skin lesions on the neck, back, chest, abdomen, both axillae and arms, buttocks, groin, and both legs in the form of prominent pustules with an erythematous base, erythematous macules and plaques, erosions, purulent crust, scales, and hyperpigmented macules. There were 'half–half' blister lesions, especially on the back (Fig. 1). The Nikolsky sign was negative. There was no mucosal involvement. The results of a direct microscopic examination of the pus on the back with Gram staining found many polymorphonuclear cells (PMN) without any bacteria. Histopathological examination showed subcorneal blisters consisting of neutrophil cells and mild acanthosis in the overlying epidermis. In the dermis, there were perivascular lymphocytes. These features supported the diagnosis of subcorneal pustular dermatosis (SPD). However, direct immunofluorescence (DIF) examination showed IgG deposits with an intercellular pattern in the epidermis , and no IgA deposits were found (Fig. 2A, B). On the basis of these findings, the patient was diagnosed with atypical PF. The patient was treated with oral cyclophosphamide 100 mg daily, which resulted in significant clinical improvement evidenced by the disappearance of all pustules, crusts, and scale lesions on the tenth day of therapy. Unfortunately, the patient was later lost to follow-up, hindering the ability to assess long-term outcomes and the sustained effectiveness of the treatment.

### Case 2

**Fig. 3:**
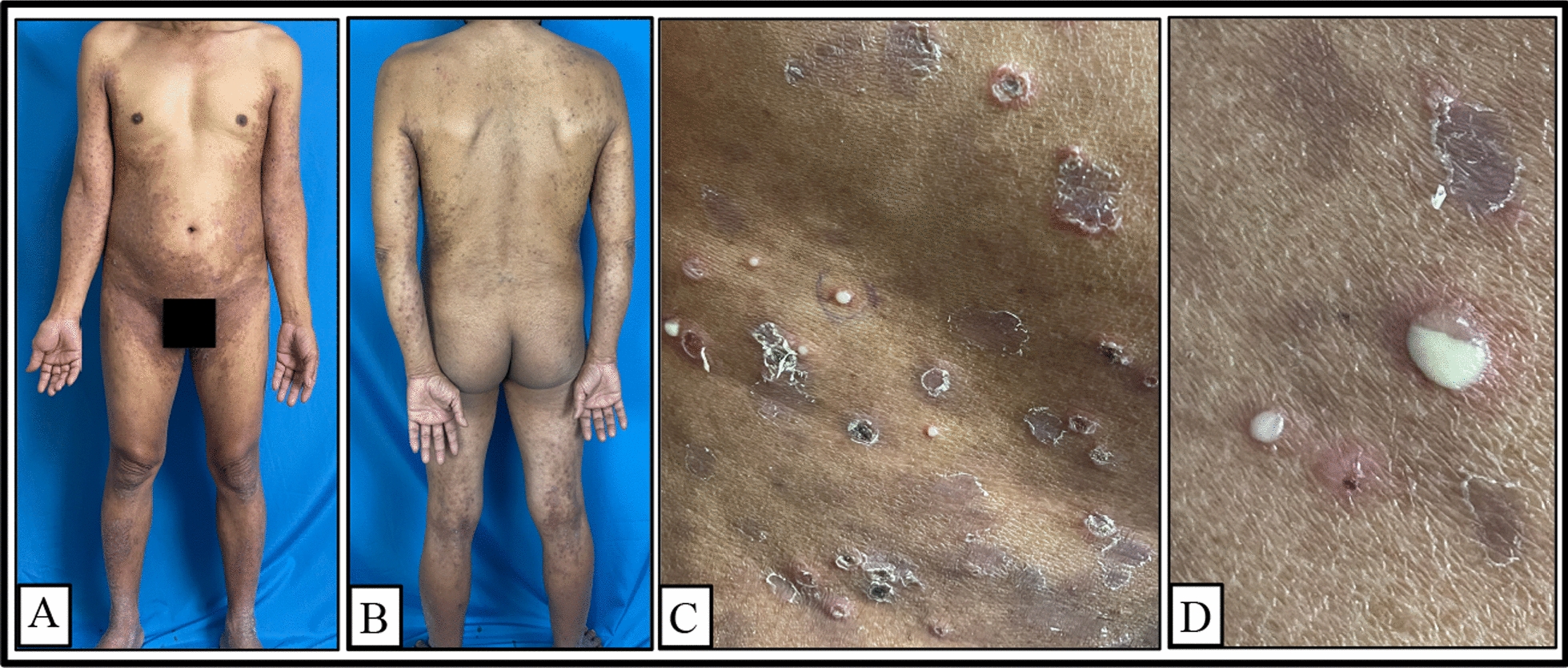
Patient 2: Skin lesions on the front view of the body (**A**), back view of the body (**B**), and back (**C**), and half–half blister lesions on the back (**D**)

**Fig. 4:**
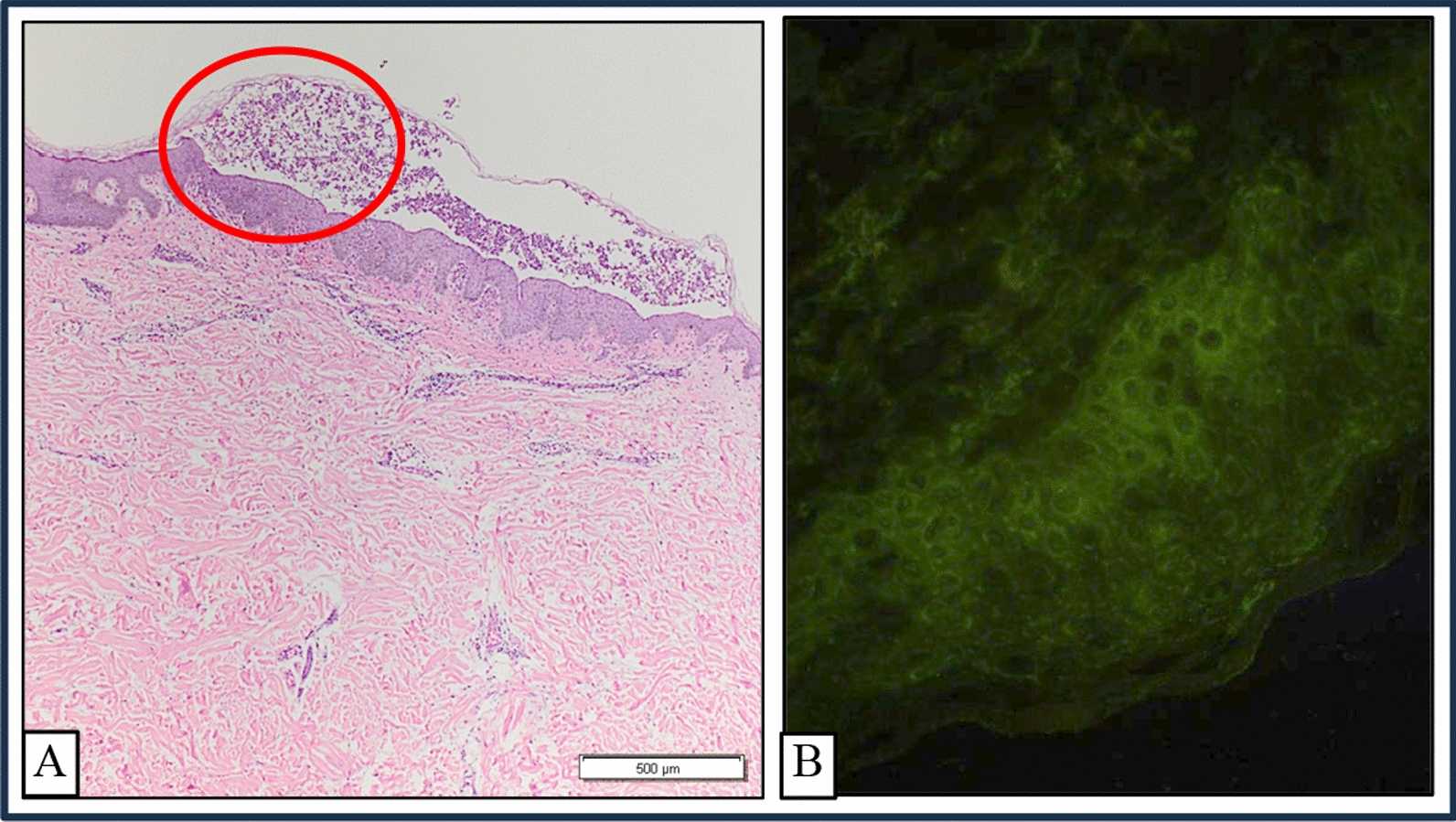
Patient 2: Histopathological examination results showing a subcorneal blister consisting of neutrophilic inflammatory cells (red circle) (**A**) and direct immunofluorescence examination results showing immunoglobulin G deposits with intercellular patterns in the epidermis (**B**)

A 57-year-old Javanese male patient presented with itchy pustules on the erythematous base, which had been present for 2 years and spread progressively, as well as a few lesions as half–half pustular blisters, which had been present for 2 weeks. The pustules broke easily to form crusts and hyperpigmented macules. He had been treated by several dermatologists and was given oral corticosteroid therapy, but it did not improve significantly. Physical examination revealed skin lesions on the neck, the back, the abdomen, both axillae and arms, the groin, and the legs in the form of prominent pustules with an erythematous base, erythematous macules, erosions, purulent crust, scales, and hyperpigmented macules. The half–half blisters were also found in this patient, especially on his back and abdomen (Fig. 3). The Nikolsky sign was negative. The direct microscopic examination of the pus on the abdomen with Gram staining showed many PMN without any bacteria. The results of histopathological examination also revealed subcorneal blisters consisting of neutrophil cells as well as neutrophil infiltration on the papillary dermis. The stroma of the connective tissue was infiltrated by inflammatory lymphocyte cells accompanied by the dilatation of blood vessels. These features supported the diagnosis of SPD. However, DIF examination also showed IgG deposits with an intercellular pattern in the epidermis layer, and no IgA deposits were found (Fig. 4A, B). On the basis of these findings, the patient was diagnosed with atypical PF. Initial sulfasalazine therapy for 87 days showed no significant improvement. However, therapy with oral azathioprine 50 mg twice daily resulted in partial clinical improvement on the 16th day of therapy. Throughout 1 year of follow-up, the patient’s skin lesions exhibited a persistent pattern of recurrence and remission, with recurrences occurring especially during stressful events. The patient eventually had significant improvement after 7 days of additional systemic corticosteroid, equivalent to prednisone 1 mg/kg/day, and psychotherapy. The patient remained free from pustules and new skin lesions during the 2-month follow-up period, with systemic corticosteroid doses being gradually tapered off without any side effects.

The patient felt that the recurrence and remission pattern of the disease was frustrating. However, the patient felt satisfied with the therapy addressing both the physical symptoms and the psychological condition and with the fact that the disease could be controlled. The patient felt optimistic and grateful for the comprehensive care.

## Discussion and conclusions

Pemphigus foliaceus is a chronic autoimmune vesiculobullous disease typically presenting as erosions, crusts, and scales on an erythematous base with burning, pain, or itching symptoms [1]. However, PF can also appear with atypical clinical manifestations, which are very rare. Several forms of atypical PF have been reported, including pustular lesions, violaceous plaques, and violaceous nodules [3–13]. Matsuo *et al*. [4] reported four atypical PF cases in 2001, identifying them as generalized pustular lesions. Similarly, Singh *et al*. [8] reported two cases of atypical PF in 2009, characterized by clinical manifestations of pustules and hypopyon bullae on the arms and upper trunk. In our case report, the patients also exhibited clinical manifestations predominantly consisting of regionally distributed pustular lesions, with some lesions appearing as half–half blisters. There have been no reports of such cases from Indonesia.

The results of histopathological examination of PF generally show intercellular vacuoles in the stratum granulosum and/or upper layer of stratum spinosum, and eosinophilic spongiosis [1]. There are only a few case reports of PF with neutrophil infiltration in the epidermis. Clinical manifestation in the form of pustular lesions, particularly half–half blister, and histopathological features of subcorneal blister with neutrophil infiltration can be found in other dermatoses, such as SPD and SPD-type IgA pemphigus [8, 9]. Both patients had clinical manifestations in the form of dominant pustular lesions with the presence of half–half blisters, as well as histopathological examination showing subcorneal blisters consisting of neutrophilic inflammatory cells, thus supporting the diagnosis of SPD.

Therefore, immunofluorescence examination is important to confirm the diagnosis. The results of DIF examination of PF showed intercellular IgG deposits on the surface of keratinocytes, and no IgA deposits were found [1]. These findings are in concordance with the DIF examination results of both patients. On the basis of clinical manifestations, histopathological, and DIF examination findings, both patients were diagnosed with atypical PF with predominant pustular lesions and neutrophil infiltration.

The treatment of PF includes topical and systemic corticosteroids, azathioprine, mycophenolate mofetil, methotrexate, cyclophosphamide, and sulfasalazine [1, 14, 15]. There is no specific therapeutic approach for PF with pustular lesions and neutrophil infiltration, because the cases are very rare; thus, atypical PF is treated with common PF therapy options [9]. Several authors have reported treatment of atypical PF with systemic corticosteroids [4–11], dapsone [5, 9, 11], azathioprine [8, 11], cyclosporine, and acitretin with good results [10]. Both patients in this case report had been given long-term corticosteroid therapy, but this was unable to control the disease. The first patient then received cyclophosphamide and showed significant improvement on the tenth day of therapy. The second patient initially received sulfasalazine 2 g/day for 87 days but showed no improvement; this patient was subsequently treated with azathioprine in combination with additional systemic corticosteroid (equivalent to prednisone 1 mg/kg/day, gradually tapered every 2 weeks) and psychotherapy, leading to controlled disease and the absence of skin lesions for 2 months of follow-up. Long-term follow-up is needed to assess long-term outcomes and the sustained effectiveness of the treatment.

In conclusion, PF can appear with atypical clinical manifestations, one of which is in the form of dominant pustular lesions with neutrophilic infiltration. Supporting examinations such as histopathology and DIF may help distinguish atypical PF from other conditions. Given the challenges in diagnosing chronic vesiculobullous diseases with unusual presentations, clinicians should include PF in the differential diagnosis when encountering prominent pustular lesions to prevent misdiagnosis and ensure proper treatment.

## Data Availability

The datasets used during the current study are available from the corresponding author on reasonable request.

## Abbreviations

DIF: Direct immunofluorescence
Ig: Immunoglobulin
PF: Pemphigus foliaceus
PMN: Polymorphonuclear cells
SPD: Subcorneal pustular dermatosis
